# Spearfishing Regulation Benefits Artisanal Fisheries: The ReGS Indicator and Its Application to a Multiple-Use Mediterranean Marine Protected Area

**DOI:** 10.1371/journal.pone.0023820

**Published:** 2011-09-23

**Authors:** Delphine Rocklin, Jean-Antoine Tomasini, Jean-Michel Culioli, Dominique Pelletier, David Mouillot

**Affiliations:** 1 Laboratoire Ecologie des Systèmes Marins Côtiers, UMR CNRS-UM2-IRD-IFREMER 5119, Université Montpellier 2, Montpellier, France; 2 Laboratoire de Biologie Halieutique – Département Sciences et Technologies Halieutiques, IFREMER, Plouzané, France; 3 Office de l'Environnement de la Corse, Corte, France; University of Western Australia, Australia

## Abstract

The development of fishing efficiency coupled with an increase of fishing effort led to the overexploitation of numerous natural marine resources. In addition to this commercial pressure, the impact of recreational activities on fish assemblages remains barely known. Here we examined the impact of spearfishing limitation on resources in a marine protected area (MPA) and the benefit it provides for the local artisanal fishery through the use of a novel indicator. We analysed trends in the fish assemblage composition using artisanal fisheries data collected in the Bonifacio Strait Natural Reserve (BSNR), a Mediterranean MPA where the spearfishing activity has been forbidden over 15% of its area. Fish species were pooled into three response groups according to their target level by spearfishing. We developed the new flexible ReGS indicator reflecting shifts in species assemblages according to the relative abundance of each response group facing external pressure. The catch per unit effort (CPUE) increased by ca. 60% in the BSNR between 2000 and 2007, while the MPA was established in 1999. The gain of CPUE strongly depended on the considered response group: for the highly targeted group, the CPUE doubled while the CPUE of the untargeted group increased by only 15.5%. The ReGS value significantly increased from 0.31 to 0.45 (on a scale between 0 and 1) in the general perimeter of this MPA while it has reached a threshold of 0.43, considered as a reference point, in the area protected from spearfishing since 1982. Our results demonstrated that limiting recreational fishing by appropriate zoning in multiple-use MPAs represents a real benefit for artisanal fisheries. More generally we showed how our new indicator may reveal a wide range of impacts on coastal ecosystems such as global change or habitat degradation.

## Introduction

The ease of accessibility to coastal marine resources coupled with considerable technical improvements in the professional fishing industry has increased the fishing pressure worldwide. This has led to an unprecedented level of exploitation [1], resulting in the collapse of many marine fish stocks [1]–[6]. Moreover, changes in exploited biological assemblages and biodiversity loss may disrupt ecosystem functioning [3], [5], [7] and then alter the sustainability of goods and services provided by ecosystems to humanity [8]–[10].

In addition to commercial fisheries, there is growing evidence of considerable yields from recreational fishing activities. For example, it is now recognised that spearfishing is one of the most frequent recreational activities in the North-West Mediterranean coastal zones [11], but is still rarely studied [12]. Indeed, evaluating and managing this activity is challenging because it is poorly organised and surveyed. More generally and at a worldwide scale, measuring the impact of the recreational fishing activities becomes even more critical since they have reached an unprecedented level overall [13], [14].

In the US, recreational fishing represents a great part of the total catches: for example, recreational catches of lingcod (*Ophiodon elongatus*) represent around 60% of the total catches, and charter activities account from 7% to 43% of the recreational fishing catches [15]. In the Mediterranean Sea, recreational activities have been evaluated to represent 10% of total fishing production [16]. Moreover, in some particular cases, the annual biomass extracted by spearfishing may reach ca. 40% of the biomass extracted by artisanal fishing [17]. In a context where marine resources range from fully exploited to overexploited [18] and artisanal fisheries are declining [19], any increase in recreational fishing effort may weaken the long-term sustainability of artisanal activities.

Marine protected areas (MPAs) were initially established for conservation issues, i.e., to protect or restore damaged ecosystems [20], [21], but they are now also considered for maintaining the marine resources [22]–[25]. They are particularly advisable for multi-specific fisheries and sedentary stocks [26], [27]. There is fair evidence that MPAs can provide higher fish biomass inside the reserve boundaries [22], [28]–[31] as well as additional resources for adjacent unprotected areas, through larval export and adults spillover [32]–[35]. In the light of fisheries management, MPA zoning can be adjusted to favour activities in some areas and restrict access to others, whether extractive or non-extractive activities. There is no evidence yet that MPAs may sustain artisanal fisheries by limiting recreational activities inside its boundaries. Here we documented this issue from a Mediterranean case study for which we demonstrated the benefit of spearfishing regulation for a local artisanal fishery. Towards this objective we developed a new flexible indicator able to disentangle the effects of various forcing factors on ecological systems by focusing on relative abundances of species response groups (RG).

In the Bonifacio Strait Natural Reserve (BSNR, France), 15% of the surface area has been closed to spearfishing since 1999 but open to artisanal fishing activities. The BSNR represents an archetypal situation for evaluating the impact of regulations targeting recreational fishing on artisanal fishery landings. We used an extensive survey of the BSNR artisanal fishery carried out from 2000 to 2007, following spearfishing prohibition.

We hypothesized that, after this prohibition, the fish usually caught by spearfishers will become available for artisanal fishers. Thus, we expect that commercial catches of species highly targeted by spearfishers will increase, but that differences in the catches of other fish species will remain marginal. To test this hypothesis we gathered fish species caught in artisanal nets into response groups according to their level of targeting by spearfishers. If spearfishing has no effect on fish assemblages, the restriction of spearfishing in the BSNR should not affect the proportions of these groups in the catches. Conversely, different trends of RG after the spearfishing restriction would imply that spearfishing had a significant impact on resources and may indirectly impact the artisanal fisheries sustainability. We then developed a new synthetic indicator including the relative abundance of the RG and describing the trends of fish assemblages after the BSNR establishment. Finally, we discussed the ability of our new flexible indicator to reveal other environmental and anthropogenic pressures on coastal species assemblages.

## Methods

### Study area

**Figure 1:**
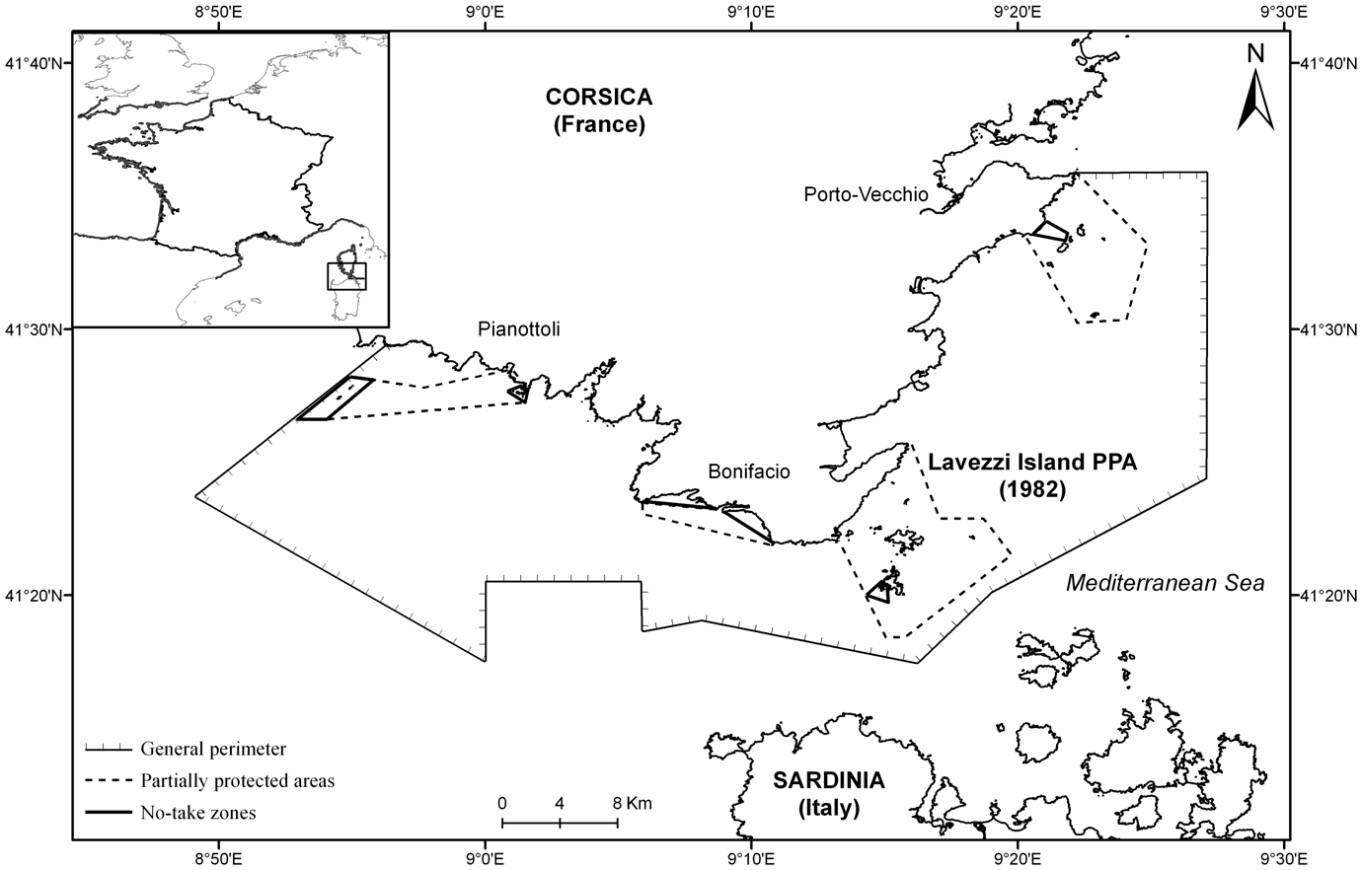
Location of the study area. The Bonifacio Strait Natural Reserve, south Corsica, France.

The BSNR, located in south Corsica in the western Mediterranean Sea (Fig. 1), has a marine surface area of ca. 800 km^2^. Its bottom substrate is predominantly covered by a mosaic habitat of rocks, sand and seagrass beds of *Posidonia oceanica* [36]. Several activities take place in this multiple-use MPA such as commercial artisanal fishing (mainly trammel nets and longlines), recreational fishing (mainly longlines, hook fishing and spearfishing), diving and sailing.

The main objective of the BSNR, created in 1999, is to protect the biodiversity, including habitats, while sustaining the local artisanal fishery. Towards these objectives, six no-take zones (NTZ) covering 1.5% of the BSNR area (12 km^2^), were created, in which all types of fishing activities, as well as diving and anchoring, are forbidden [37]. Moreover, four partially protected areas (PPA) covering 15% (120 km^2^) of the BSNR surface area, were established. In those areas, spearfishing was totally forbidden, but artisanal fishing was allowed as well as other recreational activities like small longlines, angling and reel fishing, practised from a boat or from the shore but not from islands. However, it is of importance to note that the Lavezzi Islands PPA (Fig. 1) was created in 1982, many years before the BSNR implementation. In this partially protected area, spearfishing has been totally forbidden since 1982: it is thus considered in our study as the reference zone for evaluating the performance of the BSNR multiple-use MPA.

### Fishery data and ecological survey

We used data from the artisanal fishery to evaluate the effect of the spearfishing limitation on PPAs on fish landings. The analyses were carried out on 787 fishing trips (154 in 2000, 152 in 2001, 76 in 2002, 110 in 2003, 59 in 2004, 36 in 2005, 69 in 2006 and 131 in 2007). The artisanal fishery fleet in the BSNR was composed of 13 active small boats (mean boat length 7.7 meters) working each day in the BSNR area. This activity is distributed over the whole BSNR, with four main harbours located along the coast: Pianottoli, Bonifacio, Sant'Amanza and Porto-Vecchio (Fig. 1). The mainly used fishing gears were trammel nets: those targeting fish and have a small mesh size, of 62.5 mm elongated, and those targeting lobsters, present a larger mesh size, of 125 mm elongated. To avoid a fishing gear effect [34], only trammel nets targeting fish were considered. Nets were set on the bottom at a mean depth of 33.5 m and were left underwater for 12 to 24 hours.

Data were collected in May, June and July each year from 2000 to 2007. For each fishing operation, the collected information included fishing depth, mesh size, and fishing effort, through net length and set duration. Fishing effort was thus expressed in number of pieces of trammel net (50 meters each) set per 24 hours. All caught individuals were identified at the species level and measured. Species weight was estimated using length–weight relationships [38], [39]. Catch per unit effort (CPUE) per species was calculated as the total catch in a fishing operation (in g), standardised per piece of net (of 50 m) and per day, i.e. 24 hours (CPUE in g 50 m^−1^ d^−1^).

We obtained appropriate permissions from the BSNR and from the fishermen for our observations and field study.

### Spearfishing target species and response groups

Caught species were gathered into three response groups (RG) according to their level of targeting by spearfishing. Due to the absence of published study about spearfishing in Corsica, RG were created gathering the personal knowledge of a group made of experts, scientists and spearfishers.

45 species were accounted in landings (Table 1). The first group included seven species highly targeted (HT) by spearfishing in Corsica (therefore deemed as highly sensitive to spearfishing pressure). Spearfishers target species representing a particular interest because of their emblematic value, taste, or ease of catching. Among these species some were considered as emblematic such as the brown meagre (*Sciaena umbra*), and others represented a large amount of biomass in artisanal fisheries, such as the large-scaled scorpionfish *Scorpaena scrofa*. The second group included 24 species moderately targeted (MT) by spearfishers (moderately sensitive). In this group were also species highly targeted by commercial fisheries such as the striped red mullet *Mullus surmuletus*, the common pandora *Pagellus erythrinus*, and the common dentex *Dentex dentex*. The last group included the remaining 14 species considered as never targeted (NT) by spearfishers (non-sensitive species) (Table 1). The dusky grouper *Epinephelus marginatus* was placed in the MT group as a compromise; this species is protected from spearfishing in Corsica since 1980, but some poaching still exists.

**Table 1:** Species composition of the three response groups created according to their target level by spearfishing.

Response group	Species	CPUE gain or loss (%)	p-value
**HT**	*Diplodus sargus*	357,28	<0,001
	*Labrus merula*	−42,01	<0,001
	*Labrus viridis*	150,91	0,079
	*Sciaena umbra*	218,33	<0,001
	*Scorpaena scrofa*	101,81	<0,001
	*Sparus aurata*	38,30	0,677
	*Octopus vulgaris*	28,26	<0,001
**MT**	*Conger conger*	73,13	0,067
	*Dentex dentex*	136,43	0,027
	*Diplodus puntazzo*	362,02	0,003
	*Diplodus vulgaris*	149,35	<0,001
	*Epinephelus marginatus*	184,51	0,534
	*Mullus surmuletus*	55,64	<0,001
	*Muraena helena*	−9,48	0,149
	*Pagellus erythrinus*	22,35	<0,001
	*Pagellus acarne*	1154,90	0,126
	*Pagrus pagrus*	177,52	0,101
	*Phycis phycis*	−8,79	0,009
	*Pleuronectiforms*	75,98	0,006
	*Sarda sarda*	255,77	0,053
	*Sarpa salpa*	219,61	0,506
	*Scomber sp.*	−9,45	0,458
	*Scorpaena notata*	382,61	<0,001
	*Scorpaena porcus*	4,23	0,743
	*Seriola dumerili*	111,34	0,131
	*Serranus cabrilla*	−47,99	<0,001
	*Serranus scriba*	−19,08	0,651
	*Sphyraena sphyraena*	518,81	0,006
	*Spondyliosoma cantharus*	92,59	<0,001
	*Symphodus tinca*	−3,69	0,001
	*Sepia sp.*	38,34	0,021
**NT**	*Boops boops*	154,84	0,015
	*Diplodus annularis*	−55,04	0,018
	*Labrus mixtus*	31,07	0,105
	*Lophius piscatorius*	4,27	0,604
	*Merluccius merluccius*	−80,22	0,063
	*Oblada melanura*	−34,48	0,392
	*Spicara maena*	486,48	0,002
	*Symphodus sp.*	150,00	0,001
	*Synodus saurus*	−62,50	0,219
	*Trachinus sp.*	−43,82	<0,001
	*Trachurus sp.*	11,76	0,786
	*Chelidonichthys lucernus*	17,60	0,672
	*Uranoscopus scaber*	−36,72	<0,001
	*Zeus faber*	325,27	0,047

HT: Highly Targeted species; MT: Moderately Targeted species; NT: Never Targeted species, and CPUE gain or loss (in % from 2000 to 2007) with its p-value (from the non parametric regression model).

### ReGS: A new indicator of external pressure on species assemblages

Most of the existing community-level indicators were developed for soft-bottom macro-benthic communities in coastal zones [40], whereas there is still a lack of consensus regarding the relevant ecological indicators for marine fish assemblages [41], [42]. In earlier studies, benthic species were usually gathered into ecological groups depending on their sensitivity to environmental conditions and disturbances. For instance, Borja et al. (2000) considered five ecological groups while Mistri & Munari (2008) reduced the number of groups to three (opportunistic, tolerant and sensitive species) to limit errors due to uncertainty when grouping species.

By analogy, we developed the ReGS (**Re**sponse **G**roups based on **S**ensitivity) indicator, a new simple and flexible indicator focusing on the abundance distribution among response groups differing in their sensitivity to a particular disturbance, here the spearfishing pressure.

This indicator aims to provide a synthetic information on the ecological state and trend of a whole fish assemblage facing an identified pressure. First, we considered 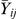 as the ratio between 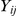 the CPUE of the response group *i* at the fishing operation *j* and the overall CPUE 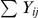 for the fishing operation *j*.
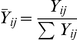
with 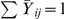


In a second step, we computed 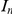 rating the CPUE of the first two groups (species highly and occasionally targeted by spearfishing, HT and MT) relative to the importance of the third group (never targeted species, NT):
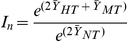



We used the exponential transformation to avoid zero values at the denominator. Similar to other authors using RG [43], we assigned larger weights to HT and NT using a factor of 2. Indeed, HT and NT have a high ecological significance because their presence or absence is representative of a particular ecological situation: the presence of HT is representative of a healthy situation (not impacted), whereas NT is representative of strong consequences of the perturbation.

**Figure 2:**
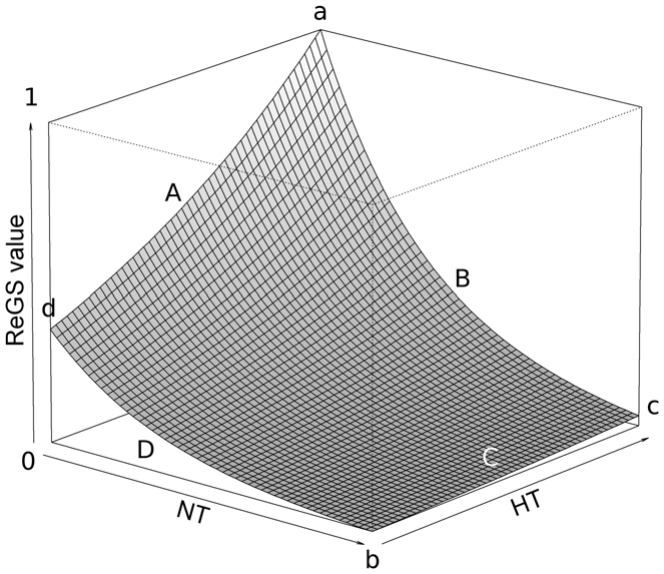
Values of the ReGS indicator according to the representativeness of the three Response Groups. X-axis: NT (species Never Targeted by spearfishing) group representativeness. Z-axis: HT (species Highly Targeted by spearfishing) group representativeness. Y-axis: ReGS value. Point a: ReGS = 1, all present species are belongs to the HT group. Point b: ReGS = 0, all species in the assemblage belongs to the NT group. Point c: ReGS = 0.119, 50% of species belongs to HT and 50% to NT. Point d: ReGS = 0.356, all species of the assemblage belong to the MT (Moderately Targeted by spearfishing) group. Line A: any individual belonging to the NT group is present. Line B: the 3 response groups (HT, MT and NT) are represented but the proportion of HT is consistently larger than the proportion of each of the two other groups. Line C: NT species are the most abundant. Line D: only MT and NT species are present.

*I_n_* was then standardised to 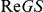
varying between 0 (only not targeted species) and 1 (only highly targeted species):
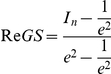



The theoretical trend of ReGS is presented in Fig. 2. Only HT and NT were used as explanatory variables since MT was deduced from the first two. The ReGS distribution pattern is asymmetrical. It decreases as the proportion of species belonging to NT increases and those belonging to HT decreases. By contrast, ReGS increases when NT proportion decreases, whereas HT proportion increases. Moreover, ReGS displays four remarkable points. Its maximum value of 1 (point a) is reached when the assemblage is only composed of species belonging to HT and the minimum value of 0 (point b) is reached when only NT species are present. An assemblage composed of 50% of species belonging to HT and 50% to NT (point c) presents a lower value (0.119), whereas an assemblage composed of only MT species (point d) has a higher value (0.356).

Boundaries of the indicator range also shed light on ReGS behaviour. Line A (Fig. 2) represents situations where any individuals of NT species are present. Line B represents situations where all groups of species are present but the proportion of HT is always larger than the proportion of each of the two other groups. Similarly, line C corresponds to the case where NT species are the most abundant. The last boundary (line D) corresponds to situations where only MT and NT species are present.

Although several combinations of abundance distribution among the RG can share the same indicator value, reducing the relative importance of NT (replaced by MT) while keeping HT fixed always results in increasing the ReGS value. Conversely, increasing HT while keeping NT fixed consistently leads to an increase of ReGS. Moreover, decreasing the proportion of NT always leads to an increase of ReGS.

### Statistical analyses

Zero values obtained for the total CPUE were considered as erroneous data due to gear malfunction and removed from the dataset [44]. Extreme values were detected using the “boxplot” method and data over the 95% confidence interval were also removed from the dataset before analyses [45].

Trends of CPUE over the study period were assessed using nonparametric regression analyses for the whole assemblage, for each response group and for species of interest showing a particular trend [46], [47], because fishing data are rarely Gaussian [48], [49] and CPUE trends are not assumed to be linear after reserve implementation [24].

This method allowed the real smoothed trend to be compared to a reference “no-effect” model. It also provided a graphical agreement between the nonparametric curve estimated from data and the null reference model. The width of the reference band was calculated based on standard errors [46]. In this study, the null hypothesis tested is “the slope of the regression is not different from 0, meaning that the CPUE of the considered group has not changed after the MPA establishment”. By contrast, the rejection of the null hypothesis means that the closure of spearfishing in the PPA had a significant impact on the catches of the considered RG.

The ReGS indicator was calculated for each fishing operation. The temporal trend of the indicator between 2000 and 2007 was assessed using a nonparametric regression analysis. All analyses were carried out using the R statistical software (http://www.r-project.org/) and the “sm.regression” smoothing method package [46], [50].

## Results

Each fishing operation displayed a mean of 0.38 caught species 50 m^−1^ d^−1^ (SE = 0.013), corresponding to ca. 11 species caught per fishing operation (SE = 0.46), and a mean CPUE of 664.5 g 50 m^−1^ d^−1^ (SE = 40.25 g 50 m^−1^ d^−1^). Landings were composed on average of 15.7 individuals p^−1^ d^−1^ (SE = 0.064 ind p^−1^ d^−1^).

### CPUE trends between 2000 and 2007

**Figure 3:**
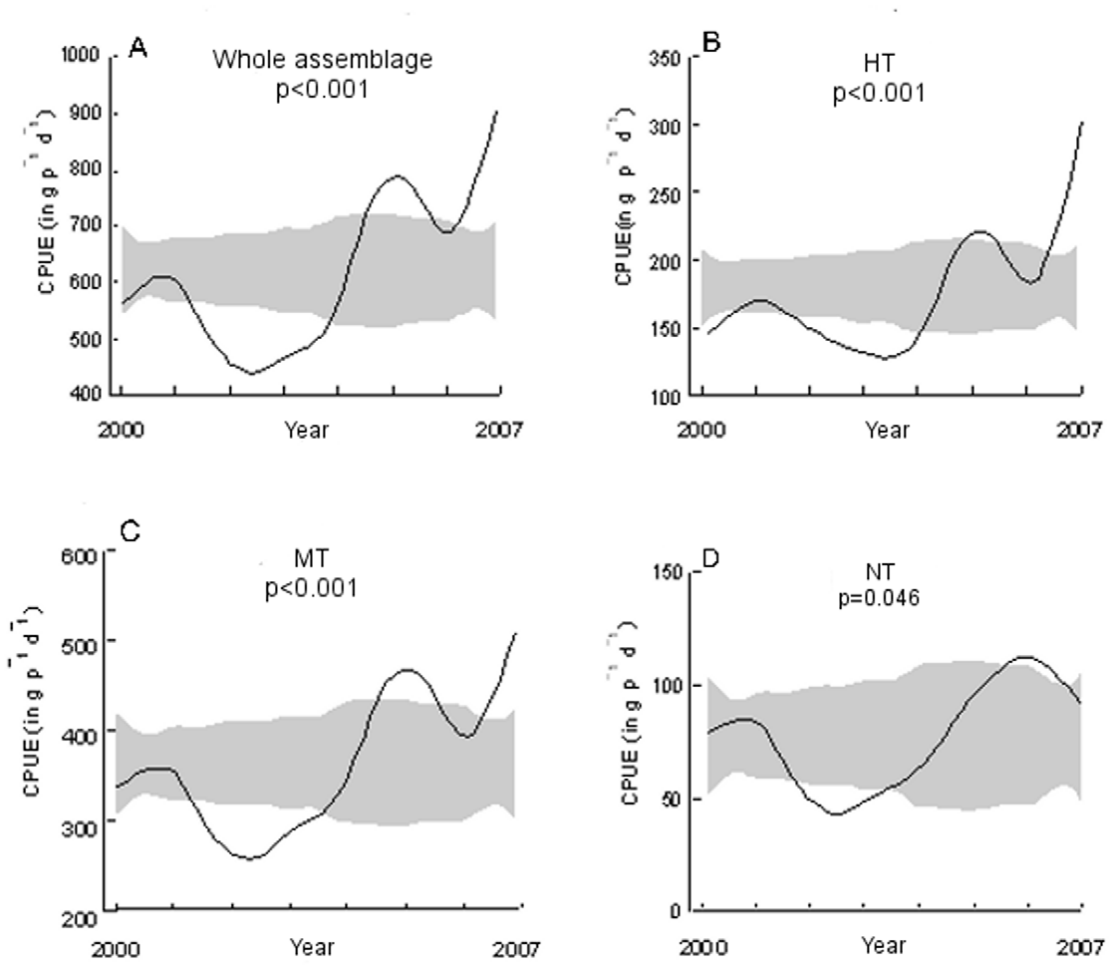
CPUE (in g 50 m^−1^ d^−1^) trends obtained with a nonparametric regression model. The grey band represents possible values for a no effect model. a) trend of the whole assemblage b) Highly Targeted (HT) species, c) Moderately Targeted (MT) species and d) Never Targeted (NT) species.

The general trend of the whole assemblage displayed a significant increase of CPUE (Fig. 3a) compared with the “no-effect” model (Table 2). We noticed that the overall CPUE increased from 606.59 g 50 m^−1^ d^−1^ to 967.14 g 50 m^−1^ d^−1^ between 2000 and 2007, corresponding to a gain of 59.4% (Table 2).

**Table 2:** Estimated slopes (gain of CPUE [in g p^−1^ d^−1^] each year), fitted values of CPUE in 2000 and 2007 and percentage increase of CPUE for the whole assemblage and each response group considering a linear regression test.

Response group	Fitted CPUE 2000	Fitted CPUE 2007	Increase 2000–2007	p-value
**Whole assemblage**	606.59	967.14	59.4%	<0.001
**HT**	157.71	318.90	102.2%	<0.001
**MT**	369.91	556.98	50%	<0.001
**NT**	78.98	91.26	15.5%	0.046

HT: Highly Targeted species; MT: Moderately Targeted species; NT: Never Targeted species. The p-value results from the nonparametric regression model.

Dividing the whole assemblage into RG disentangled the general pattern. The CPUE of each RG significantly increased over the study period, but the benefit was not equal for all groups (Fig. 3). The CPUE of the HT RG doubled between 2000 and 2007 (Fig. 3b) while the MT group showed a ca. 50% increase (Fig. 3c). NT exhibited the lowest increase:15.5% (Fig. 3d).

**Figure 4:**
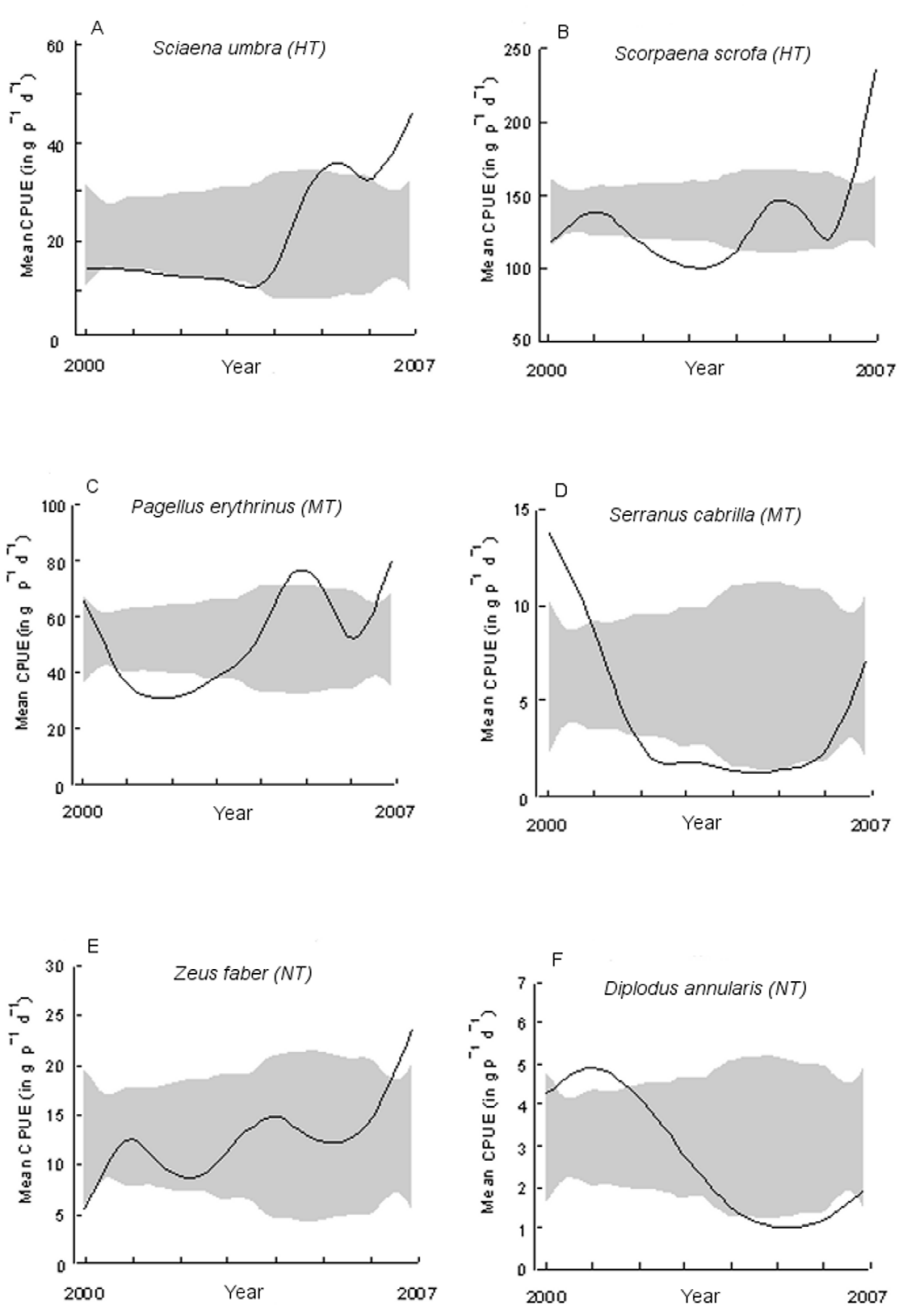
CPUE (in g 50 m^−1^ d^−1^) trends for 6 abundant species in the Bonifacio Strait Natural Reserve artisanal fishery. a) and b) *Sciaena umbra* and *Scorpaena scrofa*, from the highly targeted group; c) and d) *Pagellus erythrinus* and *Serranus cabrilla*, from the moderately targeted group; e) and f) *Zeus faber* and *Diplodus annularis*, from the never targeted group. The grey band represents possible values for a no effect model.

Some species particularly contributed to the observed CPUE trends for each response group (Table 1). For the HT group, the brown wrasse *Labrus merula* displayed a significant decrease of its CPUE while four species showed a significant increase in CPUE over time, including the brown meagre *S. umbra* (+218%, Fig. 4a) and the large-scaled scorpionfish *S. scrofa* (+102%, Fig. 4b). For the MT group, 9 species displayed a significant CPUE increase over time such as the striped red mullet *Mullus surmuletus* (+56%) and the common pandora *P. erythrinus* (+22%, Fig. 4c), whereas three species decreased, like the comber *Serranus cabrilla* (−48%, Fig. 4d). Finally, seven species out of the 14 belonging to NT showed no difference in CPUE values between 2000 and 2007. Four species displayed a significant CPUE increase such as the john dory *Zeus faber* (+325%, Fig. 4e), whereas CPUE of three species significantly decreased such as the Atlantic stargazer *Uranoscopus scaber* (−37%) and the annular seabream *Diplodus annularis* (−55%, Fig. 4f).

### Changes in the ReGS indicator

**Figure 5:**
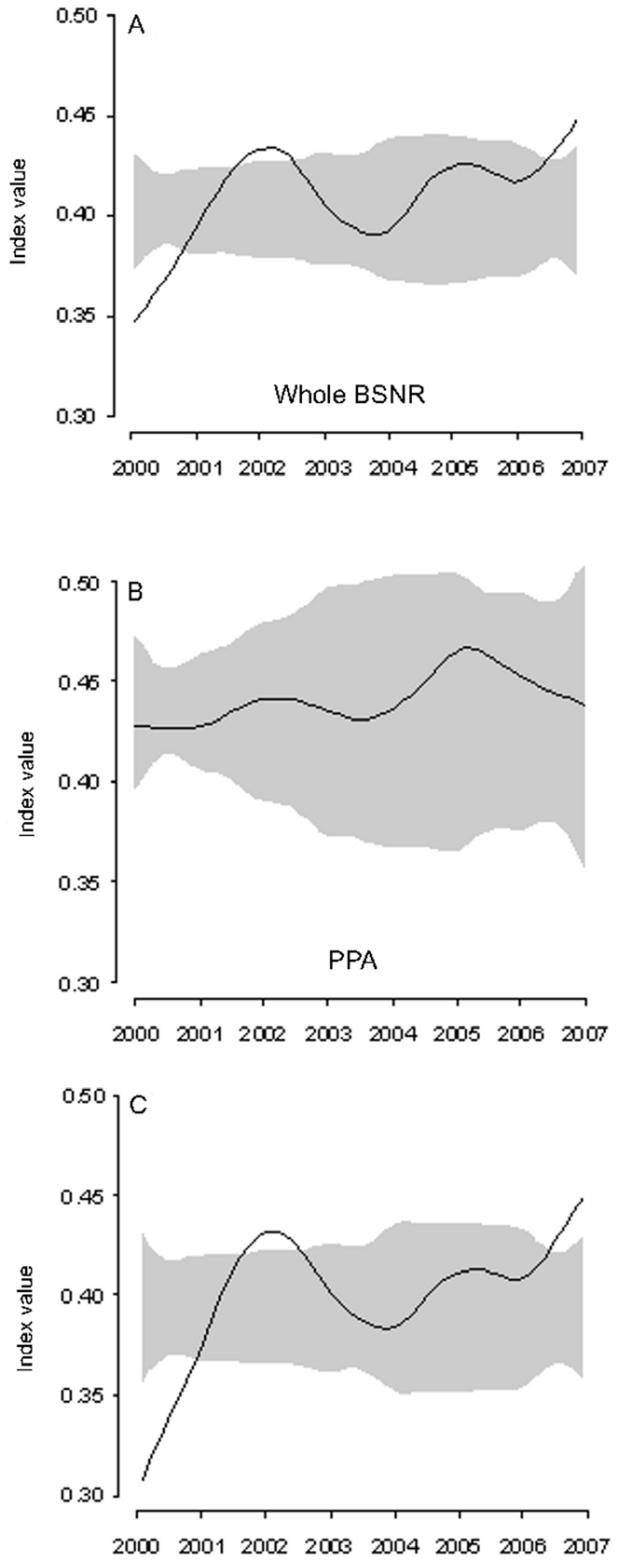
Variation of the sensitivity ReGS indicator after the spearfishing restriction. a) Whole Bonifacio Strait Natural Reserve, b) in the partially protected area (PPA) around Lavezzi Islands (protected since 1982) and c) in the reserve area outside the Lavezzi Islands reserve, protected since 1999.

In addition to significant CPUE increase for each response group, the composition of the caught assemblage itself showed marked modifications. Considering the whole reserve area, ReGS values significantly increased between 2000 and 2007 (Fig. 5a, model “no effect”, p<0.001). Its fitted values varied from 0.35 in 2000 to 0.45 in 2007. We observed an increase in the HT group representativeness over the study period (from 26.3% to 31.3% between 2000 and 2007), whereas the relative proportion of species belonging to MT and NT diminished over the same period, from 60.1% to 57.2% and from 13.6% to 11.5%, respectively.

When considering the trend of this indicator on the Lavezzi Islands area, partially protected since 1982 and thus constituting our reference are for evaluating the spearfishing impact, no significant differences of ReGS values were observed over the study period (Fig. 5b, mean = 0.43, p = 0.986). Conversely, this indicator significantly increased when considering the rest of the BSNR multiple-use MPA (exclusive of the Lavezzi Islands PPA), ReGS rising from 0.31 to 0.45 (Fig. 5c, p<0.001) between 2000 and 2007.

## Discussion

In our study, we used artisanal fisheries data to demonstrate that the closure of 15% of the MPA surface area to spearfishing was related to an overall CPUE increase of ca. 60% for artisanal fishers eight years after the BSNR implementation. MPAs effects are often assessed using underwater visual censuses (UVCs) and results mirror the impact of legislation on a subset of species assemblages [33], [51]–[54]. Cryptic and nocturnal species, apprehensive ones and overall those which are not present within transect boundaries are not thus taken into account [55]. Indeed, how can it be claimed that the fish biomass observed in UVCs will surely be available for fishers? Here, using fisheries data really allowed us to evaluate not only the ecological role of MPAs but also their socio-economic role while sustaining fisheries.

Other papers have assessed the benefits of MPAs for artisanal fisheries using experimental fishing [32] or commercial fisheries data [22], [23], [32], [34], [56]–[58]. However, such studies generally focused on spillover outside MPAs since fishing activities are usually forbidden inside MPAs [59]. In this study, we took advantage of the zoning of the fishing activities within the BSNR, where recreational fishing is regulated inside some areas, to indirectly demonstrate the significant impact of spearfishing on marine assemblages.

For that purpose, we focused on response groups, gathering species according to their sensitivity to an identified pressure. Such methodology has the advantage to drive the analysis towards a particular answer at the response group level [60]. Thus, in addition to an overall CPUE increase in artisanal catches of ca. 60% eight years after the MPA implementation, we found marked differences in the CPUE of response groups depending on the species sensitivity to spearfishing. Highly targeted species (HT) showed the highest gain of CPUE, whereas never targeted species (NT) exhibited the lowest increase. These findings are in agreement with those of Tetreault & Ambrose (2007), showing that the response of species to a MPA establishment depends on the initial fishing pressure endured by them, with highly targeted species responding more rapidly to protection [31]. It has also been showed that in a Mediterranean MPA, fish species contributing most to the biomass increase are highly catchable species [61]. Furthermore, we did not only highlight a binary response of species to protection, meaning either a rapid recovery or no difference, but we evidenced a gradient of response related to the gradient of sensitivity to spearfishing. This is in accordance with other findings underlying the significant impact of recreational activities on coastal assemblages. For instance, Westera et al. (2003) demonstrated that the line fishing only was sufficient to alter the composition of targeted fish [54]. Denny & Babcock (2004) also revealed that an area closed to commercial fishing but open to recreational fishing (such as spearfishing) is at least under the same fishing pressure as neighbouring unprotected sites [59].

While the effects of commercial fisheries on fish assemblages [62]–[65] are nowadays quite well known, the impacts of recreational activities have been until now rarely assessed due to the lack of data collection systems for non-commercial fishing [14], [66] and are still poorly known [11], [14], [17], [67]. However, recent studies showed that recreational fishing could account for more than 10% of the global fishing production [14], [16]. In the Mediterranean, catches from recreational fishing have been estimated at nearly 50% of commercial fishing production for some places [11], [17] which may challenge the sustainability of artisanal fisheries sharing the same areas and the same resources.

Ecosystems are complex and a wide set of factors, such as habitat, environmental pollution or global change, can indubitably affect the trend of all observed metrics [68], [69]. To be able to disentangle changes due to the natural variability of environmental factors from those resulting from anthropogenic pressure or management policies, other approaches have to be developed [70]. In our study, it could be argued that the CPUE increase may be due to a reduction of the fishing effort in this area. However, trammel nets used in the BSNR are passive gears and catch a large panel of species. Thus, upon decreasing net length or fishing effort, a consistent impact on each species of the whole caught assemblage should be expected whatever the RG. Our RG are built according to one pressure (spearfishing) and species belonging to the same group would unlikely be consistently driven by other forcing factors. Then we suggest that other confounding factors are of secondary importance compared to the spearfishing regulation.

At this time, other studies assessing the effect of MPAs on fish assemblages have considered species groups according to a range of distinct fishing pressures [31], [41], [71], but none has proposed an integrated indicator. The ReGS indicator was developed in the context of marine fish communities facing spearfishing pressure. In our reference area, the Lavezzi Islands, ReGS has reached a threshold at 0.43. In the other parts of the BSNR, where partially protected areas have been implemented in 1999, ReGS rapidly rose during the first three years of protection to reach a value of 0.43 in 2002, and increased up to 0.45 in 2007. This result is consistent with previous studies demonstrating that the first effect of marine reserves could be perceived within the first years of implementation [22], [24], [72]. Thus, we suggest that a fish assemblage not impacted by spearfishing pressure in South Corsica would display an indicator value ranging between 0.43 and 0.45. This could be used as a reference point for managers and be compared to other protected areas in the Mediterranean Sea, provided that they share more or less similar species as those found in the BSNR. The provision of reference points for gauging indicator values is desirable whether for fisheries management [73], [74], for assessing the MPAs performance [75] or for assessing the status of an ecosystem [73], [76], and can be considered as a target for managers [77]. Other specific points might be valuable since they have an ecological meaning. Hence, when the assemblage is highly impacted, with the opportunistic response group NT (less sensitive) representing the greater proportion of the biomass assemblage, for instance at least 50%, the indicator value cannot be higher than 0.119 (point c) (Fig. 2). Besides, an indicator having a higher value than 0.356 (point d) characterizes an assemblage where highly sensitive species are inevitably present. An increase in ReGS value is always a consequence of a higher proportion of HT and MT groups with respect to the NT group. Conversely, a decrease in ReGS is related to a larger proportion of species less sensitive to the studied pressure. We are aware that these values are idiosyncratic and cannot be considered as absolute thresholds for all Mediterranean coasts and are not relevant outside the Mediterranean. Indeed, our classification into response groups results from a consensus for the BSNR among scientists, managers and spearfishers but is not a Mediterranean classification. ReGS is flexible and each local scientist may produce his own groups according to local behaviour and legislation, and then his own thresholds.

In aquatic ecology, other several ecological indicators of disturbance have been developed in recent years, particularly those established on the concept of response groups [40], [43], gathering species according to their known or supposed sensitivity to an environmental or anthropogenic pressure [78]. Such indicators help synthesizing a set of information into a single value, providing a diagnostic about community health [79]. While the large majority of these indices were developed for benthic communities [40], few of them were dedicated to fish assemblages. The widely used indicator of biotic integrity [80] has been adapted to a range of case studies by several authors but was exclusively applied to freshwater or estuarine fish species. The FAST indicator, based on presence/absence data, and classifying species according to their size [55], has been also recently developed for coastal fish assemblages, since fish size distribution is known to be influenced by the overall exploitation level [62], [81], [82]. But one step further, our study showed that relying on response groups provided further insights into the effects of fishing on fish assemblages as it enables to disentangle the effects of various fishing pressures (commercial vs. recreational). In addition and more generally, our results showed that evaluating the MPAs effects at the assemblage level could benefit from the construction of indicators specifically related to the impact at stake, rather than attempting to control a number of forcing factors through a complex observation design.

Our new indicator ReGS is highly flexible and not exclusive to specific areas, particular taxa or particular disturbance. In the context of global warming, we may use ReGS to evaluate the changes on coastal communities simply by creating response groups according to their water temperature preferendum [83]. We may also reveal the degradation of some habitats (e.g. seagrass beds etc..) by classifying species according to their degree of requirement for these particular habitats and by using ReGS patterns across space and time.

More generally, since MPAs aim to reach different goals, at least in the Mediterranean Sea, ReGS can also cope with different targets and different requirements. If a set of species is under human pressure and may go extinct locally, then their abundance can constitute the critical point for a MPA and their managers. In this case, ReGS may be calculated by placing the set of endangered species into the most sensitive group and by increasing the weight of this group (2 in our example) to focus on the recovery of this group after practical management efforts. We dealt with abundance to characterize our response groups but, since species richness and the level of biodiversity are classical targets for MPA managers [84], we may instead consider the species richness of each response group to calculate ReGS and to reveal the positive influence of MPAs on the biodiversity of the most sensitive groups. Our indicator may be thus adapted to many MPAs management objectives by modifying weights within ReGS calculation (more or less emphasize on the most sensitive group compared to the others) or by changing relative abundances by relative (or absolute) species richness (or other biodiversity facets such as functional diversity) to measure the relative contribution of each response group. Obviously, the ReGS indicator can be applied irrespective of the working area (marine or terrestrial) and of the observation technique used, as long as species can be gathered into response groups according to their sensitivity to the considered pressure.

However, ReGS does not consider potential trophic cascades between species belonging to the same or to different groups which may blur the general observed pattern. For instance, beyond the general gain for each response group, we found a significant decrease of some species CPUE. Such trend is generally not awaited after a MPA implementation, but can be explained by trophic interactions between species through top-down effect, where an increase of predators would lead to the decrease of its preys [85], [86]. For example, the significant decrease of *S. cabrilla* could be explained by the significant increase of *D. dentex*, one of its predators (Table 1). To overcome this potential pitfall we suggest (i) to use this indicator in species rich communities to weaken the overall confounding effect of trophic cascades, (ii) to limit the number of groups to avoid groups with too few species (i.e. less than 5), or (iii) to limit its use to species assemblages with more or less similar trophic levels. These three recommendations were applied in our study case since we had 45 fish species split into three response groups with few piscivorous species. We thus consider that our results are robust regarding this critical issue.

### Conclusion

MPAs were initially developed to protect or enhance local biodiversity, but they are being more and more used as tools for fishery management. However, few studies showed that multiple-use MPAs are effective management tools for fisheries [33], [59] and, in particular, that partial regulation of recreational fishing activities can benefit artisanal fisheries. Our results demonstrated that banning a recreational fishing activity, here spearfishing, in a part of a MPA modifies the species assemblage structure and permits to promote artisanal fisheries catches [34]. There is clear evidence that recreational activities, and particularly here spearfishing, not only quantitatively impact marine resources, but in addition modify their structure. Contrary to previous studies claiming limited or no effect of partially protected areas [25], [59], we proved that multiple-use MPAs may represent effective management tools for reaching MPA goals while limiting the socio-economic impact of a total banning of fishing activities.

Evaluating the proportion of artisanal fishery yields extracted by recreational fishing activities remains a challenging issue. Hence, working in an area previously open to all types of fishing activities and then closed to spearfishing represented a great opportunity for indirectly evaluating the impact of such activities.

In the BSNR, where legislation was expected to affect biodiversity and commercial catches, the closure of only 15% of the marine coastal water surface to spearfishing resulted in a real benefit for the local artisanal fishery with a significant increase of the landings of species initially targeted by spearfishing.

Our results thus suggest that maintaining the sustainability of coastal resources and artisanal fisheries in the Mediterranean could also require more monitoring regulations on recreational activities, which compete for the same resources as artisanal fisheries. Moreover, such work is also a claim proving that regular fish catch monitoring is necessary to evaluate the real benefit of MPAs and to better understand the modifications happening among the fish communities.
